# Preoperative SARS-CoV-2 Infection Screening before Thoracic Surgery during COVID-19 Pandemic: A Multicenter Retrospective Study

**DOI:** 10.1155/2023/8993295

**Published:** 2023-03-04

**Authors:** Silvia Fiorelli, Cecilia Menna, Federico Piccioni, Gabriele Zuanetti, Franco Valenza, Marco Rispoli, Dario Amore, Monica Rocco, Erino Angelo Rendina, Mohsen Ibrahim, Domenico Massullo

**Affiliations:** ^1^Anesthesia and Intensive Care Medicine, Department of Clinical and Surgical Translational Medicine, Sapienza University of Rome, Via di Grottarossa 1035 00189, Rome, Italy; ^2^Thoracic Surgery, Department of Clinical and Surgical Translational Medicine, Sapienza University of Rome, Via di Grottarossa 1035 00189, Rome, Italy; ^3^Anesthesia and Intensive Care Unit, Azienda USL-IRCCS di Reggio Emilia, Reggio Emilia, Italy; ^4^School of Anesthesia and Intensive Care, University of Milan, Milan, Italy; ^5^Department of Anesthesia, Intensive Care and Palliative Care, Fondazione IRCCS Istituto Nazionale dei Tumori, Milan, Italy; ^6^Department of Oncology and Oncohematology, University of Milan, Milan, Italy; ^7^Anesthesia and Intensive Care, AORN dei Colli, Monaldi Hospital, Naples, Italy; ^8^Thoracic Surgery, AORN dei Colli, Monaldi Hospital, Naples, Italy

## Abstract

**Objectives:**

During coronavirus disease (COVID-19) pandemic, preoperative screening before thoracic surgery is paramount in order to protect patients and staff from undetected infections. This study aimed to determine which preoperative COVID-19 screening tool was the most effective strategy before thoracic surgery.

**Methods:**

This retrospective cohort multicenter study was performed at 3 Italian thoracic surgery centers. All adult patients scheduled for thoracic surgery procedures from 4th March until 24th April, 2020, and submitted to COVID-19 preoperative screenings were included. The primary outcome was the yield of screening of the different strategies.

**Results:**

A total of 430 screenings were performed on 275 patients; 275 anamnestic questionnaires were administered. 77 patients were screened by an anamnestic questionnaire and reverse transcriptase polymerase chain reaction (RT-PCR). 78 patients were selected to combine screening with anamnestic questionnaire and chest computed tomography (CT). The positive yield of screening using a combination of anamnestic questionnaire and RT-PCR was 7.8% (95% CI: 2.6–14.3), while using a combination of anamnestic questionnaire and chest CT was 3.8% (95% CI: 0–9). Individual yields were 1.1% (95% CI: 0–2.5) for anamnestic questionnaire, 5.2% (95% CI: 1.3–11.7) for RT-PCR, and 3.8% (95% CI: 0–9).

**Conclusions:**

The association of anamnestic questionnaire and RT-PCR is able to detect around 8 positives in 100 asymptomatic patients. This combined strategy could be a valuable preoperative SARS-CoV-2 screening tool before thoracic surgery.

## 1. Introduction

Coronavirus disease (COVID-19) pandemic has been challenging healthcare systems worldwide. Elective surgery has been suggested to be limited in order to reduce patients' traffic and to avoid virus spread [1]. Globally, at least 28 million elective operations have been deleted as a result of the first severe acute respiratory syndrome coronavirus 2 (SARS-CoV-2) pandemic waves [2]. Perioperative SARS-CoV-2 infection increases the risk of postoperative pulmonary complications and is burdened by high mortality. Mortality rate is 23.8% compared with 2% in patients without COVID-19 [3]. Despite this critical situation, there are different pathological conditions in the lung and thorax which need soonest possible surgical intervention, such as lung cancer. This condition remains the commonest cause of malignancy death worldwide and it is paramount to guarantee adequate cares and avoid unmotivated delays, protecting at the same time this frail population from an eventual contagion [4].

The role of preoperative testing for SARS-CoV-2 in surgical pathways is still not proven. Potentially, identifying presymptomatic or asymptomatic patients with undetected SARS-CoV-2 infection could help to optimize outcomes by postponing elective surgery [5], and also protect healthcare workers (HCWs) from a possible cross infection. On the other hand, evidence and universally accepted strategies are lacking, thus time and costs of screening (i.e., financial and resources) should also be considered. The widely employed screening tool to detect SARS-CoV-2 viral RNA [6] is nasopharyngeal swab test with reverse transcriptase polymerase chain reaction (RT–PCR). Some authors have also suggested thoracic computed tomography (CT) as preoperative screening, especially before major surgeries [7, 8]. The administration of an anamnestic questionnaire prior to hospitalization is a simple screening test. The questionnaire is designed to check for any history of potential SARS-CoV-2 exposure or COVID-19-related symptoms. This questionnaire enables preidentification of patients suspected of being infected with SARS-CoV-2, and thereby could help reducing cross infections of patients and HCWs [9].

The efficacy and outcome of these screening programs are still not well known. The present retrospective study assessed the results of the COVID-19 screening protocols in three different institutions before thoracic surgery in order to determine the most effective protocol with the best positive yield.

## 2. Methods

This multicenter retrospective study was approved by the Bioethics Committee of Sapienza University of Rome (no. 7008_2020), and conformed to the provisions of the Declaration of Helsinki. The study was conducted at three Italian thoracic surgical centers from March 4th to April 24th, 2020. All adult patients scheduled for any thoracic surgery and submitted to preoperative SARS-CoV-2 screening were included. Electronic medical records, anesthesia records, preoperative evaluation records, nursing records, laboratory findings, chest x-rays, and CT examinations for all patients were reviewed. Preoperative testing was defined as any test used to identify patient's SARS-CoV-2 status in the 48 hours before surgery. Testing strategies included in this analysis were as follows: anamnestic questionnaire; swab test, defined as nasopharyngeal swab and identification of viral RNA by RT-PCR; imaging by thoracic CT; combined swab test and anamnestic questionnaire; and combined thoracic CT and anamnestic questionnaire. Preoperative screening tests were requested according to local protocol.

## 3. Anamnestic Questionnaire

A COVID-19 anamnestic questionnaire was administered before hospitalization in order to detect a possible SARS-CoV-2 infection (Table 1). The items in the questionnaire are designed to check for any history of contact within the past 14 days with any confirmed COVID-19 case, a history of travel, fever (37.5°C), respiratory symptoms (coughing, sputum, and sore throat), severe muscle ache, loss of the sense of smell and/or taste, and diarrhea [10].

## 4. RT-PCR

Nasopharyngeal swab-PCR SARS-CoV-2 testing was based on the World Health Organization (WHO) protocol [11]. If SARS-CoV-2 infection was diagnosed, surgery was postponed (at least 14 days from the end of symptoms and after negative swab-PCR result).

## 5. Chest-CT

Preoperative noncontrast computed tomography (CT) of the chest was performed. Unifocal or multifocal ground-glass opacities were considered as suspicious or positive for COVID-19. In case of positive CT screening, patients underwent swab and RT-PCR to detect SARS-CoV-2 RNA.

## 6. Statistics

Screening results were presented as the number and percentage of patients with a positive screening result, with additional percentages and 95% confidence intervals (CI's), calculated using 1,000 bootstrapping samples. All statistical analysis was performed with SPSS software, version 26 (IBM Corp, Armonk, NY).

## 7. Results

**Figure 1:**
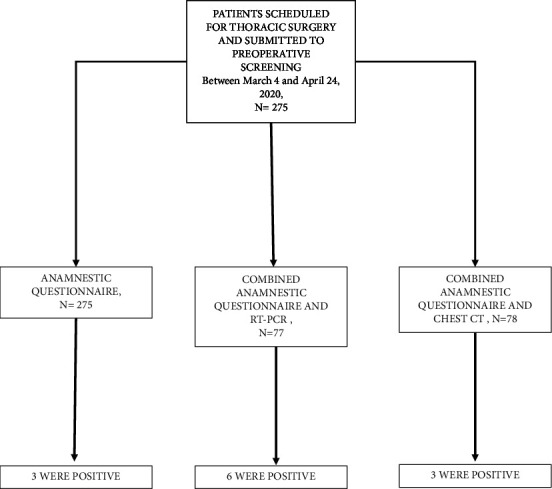
Study flowchart. CT, computed tomography; RT-PCR, reverse transcriptase polymerase chain reaction.

Between March 4th and April 24th, 2020, a total of 275 patients were scheduled for thoracic surgery procedures and submitted to preoperative screening in the participating thoracic surgery centers: 275 anamnestic questionnaires were administered, 77 patients were screened by a combination of anamnestic questionnaire and RT-PCR, and 78 patients underwent anamnestic questionnaire and chest CT according to local screening protocol (Figure 1). Demographic and clinical characteristics of patients included in the study are given in Table 2. The results for patients who underwent preoperative screening are given in Table 3. Out of 275 patients, 12 (4.3%) had positive screening results. Of these patients, three tested positive for SARS-CoV-2 using anamnestic questionnaire, four were screened using RT-PCR, while three were suspected for COVID-19 based on chest CT results.

### 7.1. Follow-Up Data

Of the three patients with a positive questionnaire, only two had infection confirmation by RT-PCR. Surgery was delayed in all patients (six) with positive screening RT-PCR; among these, only two patients developed COVID-19 symptoms. Of the three patients who had positive screening results in the chest CT, two resulted in positive RT-PCR testing, and the surgery was postponed. Among patients submitted to surgery with negative screening at questionnaire and RT-PCR, two patients developed COVID-19 symptoms within two weeks postoperatively.

## 8. Discussion

This is the first multicenter study to determine the yield of screening for COVID-19 using anamnestic questionnaire, thoracic CT, and RT-PCR in asymptomatic patients prior to elective thoracic surgery procedures. Combined preoperative screening with anamnestic questionnaire and RT-PCR demonstrated a yield of 7.8%, while combined preoperative screening with anamnestic questionnaire and chest CT demonstrated a yield of 3.8%.

During pandemic, a careful preoperative screening is becoming necessary to identify undetected SARS-CoV-2 infections. Considering that it has been reported that up to 50% of the cases of COVID-19 infection are asymptomatic or infectious in the presymptomatic phase [12], screening in nonsymptomatic patients scheduled for surgery could have a critical role.

Perioperative SARS-CoV-2 infection has been related to considerable postoperative mortality and morbidity for surgical patients [13]. Postoperative pulmonary complications occur in half of patients with SARS-CoV-2 infection and are associated with high mortality, especially in cancer or major surgery [3]. In addition, after surgery, there is a possible increased susceptibility to COVID-19 due to the proinflammatory cytokine and immunosuppressive responses to surgery and mechanical ventilation [14] and increased risk of postoperative complications and mortality [15].

During pandemic undetected infections can lead to cross infections in both patients and staff. Adequate use of personal protective equipment (PPE) during aerosol-generating procedures (AGPs), such as endotracheal intubation and airway surgical or endoscopic procedures, and a COVID-19 specific intraoperative management are paramount in order to prevent cross infections [16]. Although guidelines have been published for the management of surgical patients during the SARS-CoV-2 pandemic, so far, few studies of surgical patients have been reported, and little is known about preoperative routine screening for SARS-CoV-2 infection among surgical patients [4, 17].

Anamnestic questionnaire is a simple and rapid screening tool that has not been well investigated or standardized. A retrospective study including 2,197 consecutive patients showed that about 75% of patients with increased body temperature had positive findings in the systematic questionnaire screen for COVID-19 [18]. Although this tool can be subtly effective in identifying some symptomatic patients, it is not currently standardized, and its reproducibility is not certain [5]. The anamnestic screening could also help to anticipate a subsequent positive SARS-CoV-2 test. Certainly, the anamnestic questionnaire is at no cost while the PCR and CT scan have a high expense for the hospital. In addition, especially for mixed hospitals (with COVID- and NOT-COVID-patients), committing radiology units to perform CT scans of COVID patients delays the daily work schedule, since rooms and instruments require to be sanitized by interrupting normal activity.

As the possibility of infection transmission from asymptomatic or presymptomatic patients is currently known [12], screening of the patients merely based on the anamnestic questionnaire is no longer sufficient in high prevalence areas [19]. In this study, the anamnestic questionnaire as a sole method of COVID-19 screening resulted insufficient with the lowest positive yield (1.1%).

Nucleic-acid-based testing which is mainly RT-PCR on the nasopharyngeal swab samples is considered the gold standard for the diagnosis of to detect SARS-CoV-2 infection [5, 7]. An international cohort study involving 8,784 patients demonstrated that preoperative RT-PCR in asymptomatic patients was associated with a reduced rate of postoperative pulmonary complications, leading to significant benefit in major surgery and in high-risk areas. However, a relevant proportion of patients are false-negative at test (2–29%) [20], and the diagnostic accuracy remains challenging. RT-PCR sensitivity and accuracy are affected by several factors, including the type of samples, inadequate specimen quality, the timing of collection (too early or too late), sampling methods, training of operators, specimens improperly handled or transported, and quality of detection reagent or RT-PCR instrument [21]. Despite presenting the factors which may affect the result of the RT-PCR, and also considering the modest positive predictive value of RT-PCR in asymptomatic patients, it would still be advantageous to postpone the surgery or repeat the test (in case of emergency needs to the surgery) [19, 22]. Conversely, in low-risk countries, testing costs and procedural delays outweigh the benefits of screening. In fact, in populations with a low disease prevalence (<2%), RT-PCR screening failed to prove its advantages in clinical practice [23]. In these situations, screening can be more powerful with a symptom questionnaire. An additional screening with PCR testing in high-risk patient groups could be considered [24] in order to improve the positive yield, as demonstrated in this study (7.8% with combined screening vs. 5.2% with RT-PCR only).

Another screening tool for COVID-19 that has been proposed and hotly debated is thoracic CT [25]. CT could be feasible as a screening since patients may have abnormalities on chest imaging before the onset of symptoms. The potential value and advantage of CT is that it is widely available and rapid. During early stage of pandemic some studies reported that CT has high sensitivity and specificity in the evaluation of suspected COVID-19 pneumonia [26, 27]. A more recent meta-analysis, however, showed that in a low disease prevalence area the positive predictive value of CT scan can be up to ten times lower than that of RT-PCR, related to a low CT specificity [28]. Several studies confirmed that in contrast to the high sensitivity (93–98%), chest CT scan has a low specificity in detecting COVID-19 pulmonary alterations in symptomatic patients (25–71%) [26, 27, 29]. When used as a screening tool in identifying COVID-19, CT scan demonstrated a sensitivity of 68.4% and specificity of 88% [30]. According to this study, several reports showed no additional benefit in adding CT scan to other screenings such as anamnestic questionnaire and RT-PCR [5, 7, 24, 31], and a low positive yield in asymptomatic preoperative patients [7, 8, 32, 33]. In particular, the SCOUT multicentric study demonstrated a positive yield of 0.7% (95%CI: 0.2–1.1) for chest CT scan, 1.1% (95%CI: 0.6–1.7) for RT-PCR, and 1.5% (95%CI: 0.8–2) using a combination of chest CT scan and RT-PCR, highlighting the limited value of adding chest CT scan to RT-PCR screening [7]. It can be suggested that currently chest CT scan has no additional value in the preoperative screening of asymptomatic patients.

Considering pandemic in Italy, on April 7th, 2020, the number of notified COVID-19 cases in Italy was above 135,000. The epidemic was disproportionately hitting some northern areas, with a prevalence that varied among Italian regions from 11.2% in the Lombardy region, to 0.57% and 0.53% in Lazio and Campania region, respectively [34]. Despite the differences in prevalence among the different centers, the positive yield of the CT scan was not increased in the area with the highest prevalence [34].

The current study has some limitations. First, patients undergoing preoperative screening were included retrospectively. Second, the study was conducted during the lockdown period in Italy, which effectively started on March 9th, 2020. Consequently, a decreasing prevalence during the inclusion period may have led to a decreasing yield. Third, another limit is the small sample size of the study. Finally, no further analysis for risk factors could be performed due to the limited number of patients with positive results. Moreover, the present study population consisted of surgical patients and thus was not fully representative for the general population. Finally, bias related to the employed screening tool, bias from missing data, and selection bias of reported outcomes could not be excluded.

## 9. Conclusion

This study evaluated preoperative COVID‐19 screening in the early pandemic in thoracic surgery. Using a combination of anamnestic questionnaire and RT-PCR as preoperative scrrening tool demonstrated to be able to properly detect infected patients with SARS-CoV-2 in almost 8 in every 100 asymptomatic patients. Another combination, anamnestic questionnaire and chest CT scan, would have a lower value in detecting the infected persons. Therefore, due to its simplicity and feasibility, all patients can be screened for symptoms before presenting to the hospital through an anamnestic questionnaire. Indeed, further research is needed to identify and standardize the optimal preoperative screening strategy applicable to different societal context.

## Figures and Tables

**Table 1:** COVID-19 anamnestic questionnaire.

COVID-19 anamnestic questionnaire
Any risk of potential SARS-CoV-2 exposure, including
(1) Close contact with a confirmed case of COVID-19 in the past 14 days
(2) Close contact with someone who displays symptoms of anosmia or hyposmia (loss of smell), ageusia or hypogeusia (loss of taste), cough, sore throa,t or dyspnea in the past 14 days
(3) Overseas or interstate travel in the past 14 days, either by plane or cruise ship
(4) Provenience from a high-risk area

*Presence of symptoms such as*
(1) Fever
(2) Cough
(3) Sore throat
(4) Burning eyes
(5) Widespread pain
(6) Dyspnea
(7) Asthenia
(8) Anosmia/hyposmia
(9) Ageusia/hypogeusia
(10) Diarrhea

**Table 2:** Clinical characteristics of patients undergoing preoperative screening.

Characteristics	Anamnestic questionnaire (*N* = 275)	Combined anamnestic questionnaire and SARS-CoV-2 RT-PCR (*N* = 77)	Combined anamnestic questionnaire and chest CT scan (*N* = 78)
Age (years ± SD)	63.22 ± 13.26	64.66 ± 13.42	61.84 ± 13.60
Sex (M/F)	163/112	52/25	40/38
Hypertension (yes/no)	128/147	48/29	26/51
Diabetes mellitus (yes/no)	28/247	11/66	3/75
CAD (yes/no)	20/255	13/64	6/72
COPD (yes/no)	104/175	19/58	10/68
ASA II/III	120/155	34/44	58/20

*Thoracic surgery center*			
SAND	138	68	0
ITM	78	9	78
MON	59	0	0

*Scheduled surgery*			
Lung resection open	89	43	32
Lung resection VATS	122	11	29
VATS/pleural biopsy	23	6	8
Mediastinoscopy	16	7	4
Mediastinotomy	10	4	3
Tracheal surgery	5	5	0
Chest wall resection	3	0	2
Rigid bronchoscopy	7	2	0

The data are expressed as the mean and SD or N° of patients. Abbreviations: ASA, American Society of Anesthesiologists; CAD, coronary artery disease; COPD, chronic obstructive pulmonary disease; F, female; CT, computed tomography; ITM, Fondazione IRCCS Istituto Nazionale dei Tumori, Milan, Italy; M, male; MON, A.O. dei Colli, Ospedale Monaldi, Napoli, Italy; RT–PCR, reverse transcriptase polymerase chain reaction; SAND, Sant'Andrea Hospital, University of Rome “Sapienza”, Rome, Italy; SD, standard deviation; and VATS, video-assisted thoracoscopic surgery.

**Table 3:** Results of screening with anamnestic questionnaire, combined anamnestic questionnaire, and SARS-CoV-2 RT-PCR, combined anamnestic questionnaire and chest CT.

	*Preoperative COVID-19 screening*				
	Anamnestic questionnaire	SARS-CoV-2 RT-PCR	Chest CT scan	Combined anamnestic questionnaire and SARS-CoV-2 RT-PCR	Combined anamnestic questionnaire and chest CT scan
Positive screening result	3/275	4/77	3/78	6/77	3/78
No./total no.^*∗*^ % (95% CI)^#^	1.1% (0–2.5)	5.2% (1.3–11.7)	3.8% (0–9)	7.8% (2.6–14.3)	3.8% (0–9)

Abbreviations: CI, confidence interval; CT, computed tomography; RT-PCR, reverse transcriptase polymerase chain reaction. ^*∗*^A positive chest CT result was defined as Unifocal or multifocal ground-glass opacities were considered as suspicious or positive for COVID-19. # A 95% confidence interval was calculated based on 1000 bootstrap samples.

## Data Availability

The data used to support the findings of this study are available upon request (silvia.fiorelli@uniroma1.it).

## Abbreviations

AGPs: Aerosol-generating procedures
CIs: Confidence intervals
COVID-19: Coronavirus disease
CT: Computed tomography
DLTs: Double-lumen tubes
HCWs: Healthcare workers
PPE: Personal protective equipment
RT–PCR: Reverse transcriptase polymerase chain reaction
SARS-CoV-2: Severe acute respiratory syndrome coronavirus 2
WHO: World Health Organization.
